# Supporting the vulnerable: developing a strategic community mental health response to the COVID-19 pandemic

**DOI:** 10.1177/1039856220944701

**Published:** 2020-07-30

**Authors:** Sumana Thomson, Trung Doan, Dennis Liu, Klaus Oliver Schubert, Julian Toh, Mark A Boyd, Cherrie Galletly

**Affiliations:** Northern Adelaide Local Health Network, Australia; Adelaide Medical School, Faculty of Health and Medical Sciences, The University of Adelaide, Australia; Northern Adelaide Local Health Network, Australia; Northern Adelaide Local Health Network, Australia; Adelaide Medical School, Faculty of Health and Medical Sciences, The University of Adelaide, Australia; Northern Adelaide Local Health Network, Australia; Adelaide Medical School, Faculty of Health and Medical Sciences, The University of Adelaide, Australia; Northern Adelaide Local Health Network, Australia; Northern Adelaide Local Health Network, Australia; Infectious Disease Medicine, Adelaide Medical School, Faculty of Health and Medical Sciences, The University of Adelaide, Australia; Northern Adelaide Local Health Network, Australia; Medical Specialties, Adelaide Medical School, Faculty of Health and Medical Sciences, The University of Adelaide, Australia; Ramsay Health Care (SA) Mental Health Services, Australia

**Keywords:** public health, COVID-19, pandemic, schizophrenia, mental health

## Abstract

**Objectives:**

The COVID-19 pandemic poses significant risks to the vulnerable patient population supported by community mental health (CMH) teams in South Australia. This paper describes a plan developed to understand and mitigate these risks.

**Methods:**

Public health and psychiatric literature was reviewed and clinicians in CMH teams and infectious disease were consulted. Key risks posed by COVID-19 to CMH patients were identified and mitigation plans were prepared.

**Results:**

A public health response plan for CMH teams was developed to support vulnerable individuals and respond to the COVID-19 pandemic. This plan will be reviewed regularly to respond to changes in public health recommendations, research findings and feedback from patients and clinicians.

**Conclusions:**

The strategic response plan developed to address risks to vulnerable patients from COVID-19 can assist other CMH services in managing the COVID-19 pandemic.

Coronavirus disease 2019 (COVID-19) is an infectious disease caused by severe acute respiratory syndrome coronavirus 2 (SARS-CoV-2). First identified in December 2019, COVID-19 was declared a pandemic by the World Health Organisation (WHO) in March 2020.^1^ Individuals with cardiovascular disease, uncontrolled hypertension, diabetes and respiratory disease have been found to be more vulnerable, with increased risk of respiratory complications and death.^2^

Since the first reported case in Australia on 25 January 2020, significant efforts have been made on state, national and international levels to limit the spread and impact of the illness. These include travel bans and social distancing, with a reduction in cases but widespread effects on community functioning. Significant psychological consequences of the pandemic and resultant social upheaval require attention, particularly for the most vulnerable.

Community mental health (CMH) teams in the Northern Adelaide Local Health Network (NALHN) serve a highly vulnerable patient population. In 2016, the City of Playford was ranked as one of the most disadvantaged urban local government areas in Australia.^3^ Determinants of poor mental and physical health include poor educational attainment, high unemployment, financial insecurity, mortgage and rental stress, over- and under-nutrition, unsafe neighbourhoods, and high rates of domestic violence and drug and alcohol abuse.^4^ Socio-economically patterned disease cascades^5^ are evident, with the outcomes of transgenerational trauma and social marginalisation seen in levels of psychiatric morbidity and 10-year life expectancy gap compared to those living in Adelaide’s least disadvantaged suburbs.^6^

The majority of people supported by the CMH service have diagnoses of chronic schizophrenia and other serious mental illnesses (SMI) including major affective disorders and borderline personality disorder, often complicated by comorbid substance abuse and physical comorbidity. Primary mental health disorders increase the risk of infections including pneumonia.^7^ This has been linked to the use of medications increasing cardiometabolic risk,^8^ high prevalence of smoking^9^ and substance abuse,^10^ and higher rates of medical comorbidities such as obesity, diabetes, cancer and cardiovascular disease.^11^ Clozapine, in particular, is associated with pneumonia secondary to sialorrhea and risk of aspiration.^12^ These factors increase the risk of more serious illness and mortality from COVID-19.

In addition to direct medical risk, there is concern about the ability of people with SMI to engage in public health measures. Cognitive impairment, a core feature of schizophrenia, can affect the ability to understand and think critically about information, which may lead to difficulty adhering to health advice. Disorganised behaviour may increase risk of disease transmission. Positive symptoms of schizophrenia may be exacerbated, with social distancing, increased isolation and conspiracy theories leading to increased distress and anxiety through intensified delusional beliefs.

Negative symptoms of schizophrenia may lead to neglect of physical health and protective measures, and lack of insight poses another challenge through mistrust of services. However, Maguire et al.^13^ found people with schizophrenia reported willingness to adopt protective measures during the 2009 H1N1 pandemic, although at lower rates compared to the general population. A study of risk perception in people with schizophrenia found that some degree of anxiety may increase likelihood of considering protective measures, but too high a level of concurrent anxiety resulted in individuals feeling less likely to utilise meaures.^14^ Engagement and education is therefore critical.

It has been suggested that pre-existing mental health conditions would increase the risk of psychological distress during the pandemic.^15^ Changes in routine and psychosocial stressors could increase the risk of relapse in bipolar disorder and psychotic disorders, exacerbate anxiety disorders such as illness anxiety and obsessive compulsive disorder, and precipitate situational crises in those with underlying personality vulnerabilities.

This is particularly concerning in the face of social change and significant decline in the economy. In 2016, youth unemployment in the City of Playford was 23.5%, with some of its suburbs recording rates as high as 50%–60%,^16^ further compounded by the 2017 closure of car-maker Holden’s local manufacturing site. In the wake of COVID-19, mass scale job loss, particularly of younger casual workers, is likely to worsen conditions further with implications for mental health and suicide risk.^17^ People with SMI are likely to be rendered more vulnerable, further increasing their risk of homelessness,^18^ an additional challenge for follow-up, risk of transmission and mortality.

Finally, the direct and indirect impacts on mental health of COVID-19 are unclear, both in the short term and long term. Elevated levels of depression, anxiety and post-traumatic symptoms have been found a year after exposure to SARS^19^ and quarantine has been linked to post-traumatic stress symptoms, confusion and anger.^20^ For others, such as those with social anxiety, an alleviation of usual pressures may bring short-term relief. There is much still to be learnt about the neuropsychiatric sequalae of COVID-19 infection, with consideration given to the impact infection and treatment may have on precipitating or exacerbating psychosis.^21,22^ For those already affected by SMI, the impacts of further uncertainty and loss may be especially profound.

The aim of this paper was to highlight some of these considerations and the strategies put in place in CMH teams in NALHN to manage patient care during the pandemic, in the context of significant ongoing community disadvantage.

## Methods

The NALHN Mental Health Service has a catchment population of 350,000, with three CMH teams supporting the region.

As COVID-19 cases began to rise in Adelaide, health service management, consultant psychiatrists and CMH team leaders worked together to develop a plan to manage the risks of this vulnerable population. Potential concerns and responses were collated, relevant public health and psychiatric literature were reviewed, and an infectious disease specialist (MB) was consulted. A summary of recommendations was devised.

## Results

**Figure 1.**
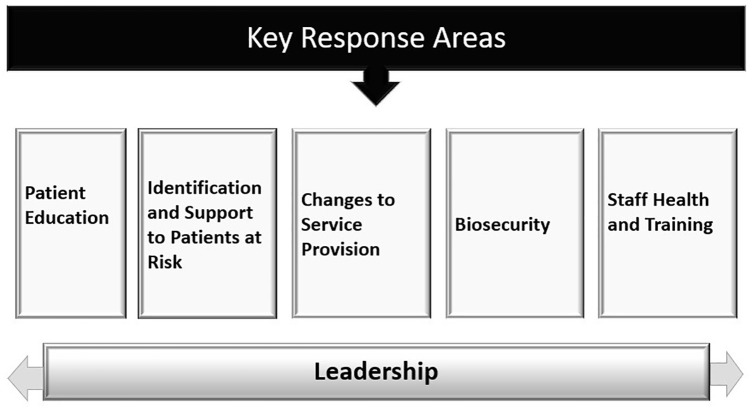
Key response areas.

A public health response document was developed, identifying five key areas to support vulnerable individuals during the COVID-19 pandemic (Figure 1).

**Figure 2.**
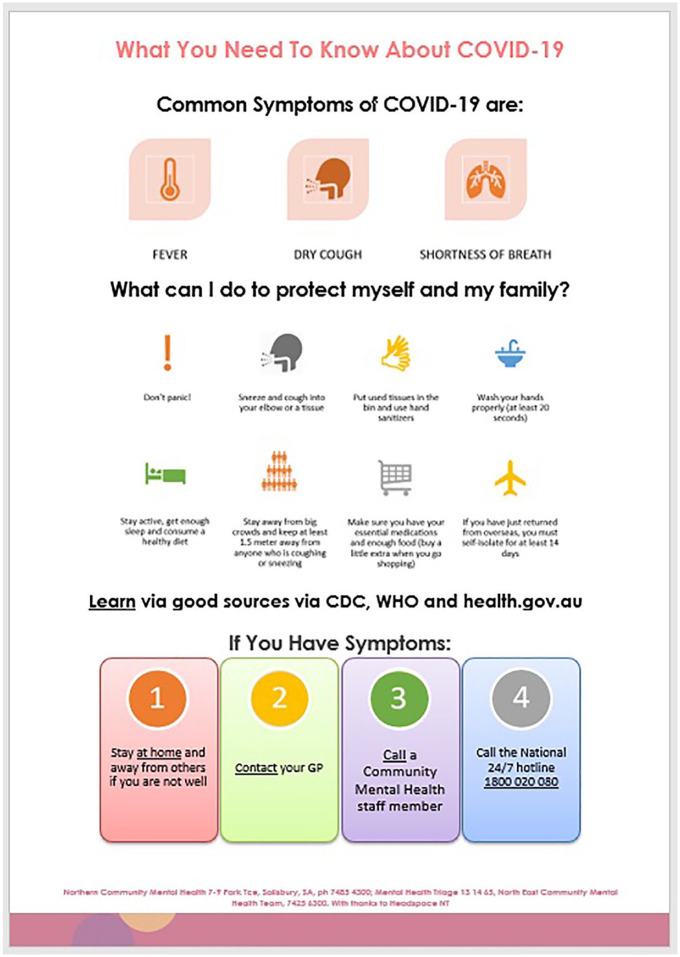
COVID-19 patient information pamphlet.

To provide accessible and consistent health messaging, educational strategies for patients were implemented (Table 1) and a pamphlet was adapted from Anglicare, drawn from SA Health and WHO recommendations (Figure 2).

**Table 1.** Patient education

**Written information** 1. Identify or create information pamphlets on COVID-19 symptoms, relevant call lines, social distancing requirements and hand hygiene in a range of formats and accessibility options including suitable for patients with lower health literacy, different languages, larger font and braille 2. Identify and promote reliable sources of information for patients e.g. the SA Health dashboard (https://www.sahealth.sa.gov.au/wps/wcm/connect/public+content/sa+health+internet/conditions/infectious+diseases/covid+2019) **Dissemination of information** 1. Display and hand out information and hand sanitiser in CMH waiting rooms and interview rooms 2. Offer support onsite via waiting room ‘concierge’, a clinician providing information to all visitors and staff upon entry 3. Offer demonstrations of good hand hygiene and support patients to learn this, including videos in the waiting room with support from concierge clinician 4. Post out pamphlets with hand sanitiser to patients 5. Care coordinators to bring written information and hand sanitiser with them to face-to-face visits when occurring **Verbal information** 1. CMH clinicians to provide verbal information and support on tele-health and face-to-face contacts, as well as dispel myths 2. Provide information regarding self-care (hygiene, sleep, maintaining and optimising physical and mental health, addressing anxiety) **Cultural support** 1. Provide referral to and liaison with cultural supports such as Aboriginal Cultural Healing team and Ngangkari healers to engage with patients about their health and holistic care

Identification of and support to vulnerable patients was key (Table 2). Vulnerable patients were identified based on:

(i) Medical vulnerability due to chronic medical comorbidities. An alert on the medical record was adopted to flag and monitor those deemed to be at medical risk from COVID-19;
(ii) Socio-economic and psychiatric vulnerability (homelessness, significant functional impairment, unstable psychiatric illness).

**Table 2.** Identification and support of patients at risk of COVID-19 complications

**Identification of risk and formation of individual management plan** 1. Identify and flag patients at risk at clinical reviews and handovers on the basis of medical, socio-economic and psychiatric vulnerability 2. Discuss with multidisciplinary team (MDT) and line manager 3. Ensure MDT formation of a COVID-19 support plan 4. Maintain close monitoring of mental and physical health (on face-to-face reviews if scheduled; if required medical review triage and link in with general practitioner (GP) or liaise with emergency department) 5. Increase contact during high-risk periods for individual patients (such as concurrent medical illness, recent medical admission, increased social adversity, recent homelessness, family conflict, substance use or worsening of mental state) **Connect those at risk with health supports** 1. Ensure ongoing GP care and, if indicated, specialist care 2. Assist patients to form/revise their health care plan(s) with their GP 3. Encourage influenza vaccine and support to access government-funded vaccine if eligible due to chronic disease 4. Ensure reliable provision of medications. **Connect those at risk with social supports** 1. Identify risk (risk of homelessness, state of the home, food security, supplies, domestic violence) 2. Link in with social services such as COVID-19 support phone lines for those in self-isolation 3. Explore and support need for food/basic necessities for those without supports. Consider support funding via SA Health or link in to other appropriate services (such as non-government agencies) 4. Provide support for those at risk of or experiencing unstable accommodation and homelessness via social work in care coordination and/or link in to and liaison with non-government agencies 5. Ensure specific registration in electronic patient file and escalation in MDT, liaison with domestic violence supports and police (Family Safety Framework) for suspected or confirmed domestic violence 6. Extend cultural supports with community (via videoconference, phone) to reduce social isolation while maintaining social distancing; link in with support agencies and workers such as Aboriginal Liaison Officers **Ongoing evaluation and re-evaluation** 1. Re-evaluate ongoing need/risk at clinical review 2. Conduct regular senior CMH team clinician meetings/briefings (via videoconference with other sites and levels of leadership) to ensure strategies remain consistent and current

Strategies including development of individual plans and connection to health and social supports were implemented. Changes to service provision were required to meet the challenges of the pandemic, including use of tele-health and specific COVID-related considerations for outpatient programmes and home visits (Table 3). Patients with an increased risk of transmitting COVID-19 were also identified and strategies were formed to manage these risks (Table 4).

**Table 3.** Changes to service provision

**Service schedule** 1. Reschedule non-essential or non-urgent face-to-face appointments and OPDs where appropriate and acceptable to the patient to help minimise risk and help maintain social distancing 2. Immediately suspend all group therapies in keeping with social distancing requirements. Care coordinators or group therapists to make contact with clients by phone to provide support while tele-health options (group or individual) are arranged where possible 3. Move day community psychosocial programmes (Club 84 and the Gully) to videoconference platform to support ongoing social engagement **Tele-health** 1. Conduct service-level evaluation of tele-health options for assessment, service delivery and support (e.g. HealthDirect or another agreed and secure platform) 2. Where possible and appropriate, use tele-health and phone contacts 2.1 Do case-by-case review of appropriateness and usefulness of the tele-health platform for individual patients, as this requires the patient to have a reliable phone or computer; judgement of affect and rapport may be limited and distress may be more difficult to manage remotely 2.2 If patient has no access to telephone or internet connection and tele-health is considered appropriate, explore access options via next of kin or family: investigate if patient can be supported to access to technology via support funding and non-government organisations 2.3 If patient has no access to telephone or internet connection, use face-to-face assessment. Alternatively, to maintain social distancing by minimising number of people in a room, consider combined face-to-face and videoconference consult, with one or more staff visiting face-to-face and another attending via videoconference on staff member’s phone 2.4 Adhere to Did Not Attend (DNA) protocol if patient does not answer scheduled consult or cannot be reached: attempt contact twice, contact next of kin, perform risk assessment, document and discuss with line manager and care coordinator to arrange follow-up call, face-to-face visit and/or liaison with other members of care team such as GP **Clozapine clinics** 1. Rationalise reviews considering the level of medical comorbidity and risks of accessing clinic in line with current Office of the Chief Psychiatrist (OCP) and Clozaril Patient Monitoring System (CPMS) guidelines (currently recommended: face-to-face contact with clinical staff reduced from monthly to two-monthly). Continue usual investigations as per protocol and re-evaluate as new guidelines emerge. If the patient has been on clozapine long term (greater than 1 year) continuously, with nil history of neutropaenia there may be a rationale for the frequency of blood tests to be reduced further during the pandemic as peak incidence of neutropaenia is within the first few months of starting therapy and is negligible after 1 year, but this is undergoing further evaluation and has not yet been formally incorporated into practice guidelines.^12^ **Depot clinics** 1. Review alternative service delivery (such as home visiting) with risk assessment to determine appropriateness on a case-by-case basis **Changes to process for home visiting** 1. Make phone calls prior to visits to ascertain presence of illness at the home via Community Based Information System (CBIS) Screening questions 2. Use the CBIS Novel Respiratory Pathogen Screening Tool checklist during phone and remote contacts and prior to face-to-face contacts to screen for symptoms of COVID-19 (with mandatory questions of: cough, sore throat, headache, fever or history of fever, shortness of breath, diarrhoea), medical comorbidities (diabetes, cardiovascular disease, respiratory disease, obesity, renal disease), travel history (within last 14 days, domestic or international to a region with sustained human-to-human transmission or outbreak), exposure (contact with confirmed case) and any COVID-19 testing results 3. If patients develop symptoms suggestive of COVID-19 (self-reported, detected on clinical encounter on history or on screening), facilitate testing and appropriate care **Use of personal protective equipment (PPE)** 1. Use personal protective equipment (PPE) during face-to-face contacts in accordance with SA Health Policy^23^ 2. When seeing a patient not suspected of having COVID-19, observe social distancing (1.5 m) unless unavoidable (e.g. physical examination), practice hand hygiene, with no requirement for PPE 3. Attention to travel alerts: if a patient has epidemiological risk factors such as travelled overseas in the last 14 days, interstate in the last 7 days or other relevant known risks at time of review, delay team assessment if appointment until after the 14-day quarantine period. If unable to reschedule appointment, clinical staff to wear PPE (surgical mask, protective eyewear, long sleeve gown and gloves) when assessing the patient 4. When seeing a symptomatic patient with confirmed or unconfirmed COVID-19, undertake contact and droplet precautions (gown, gloves and eye protection for the clinician and mask for both staff and the patient)

**Table 4.** Biosecurity

**Identification of patients at risk of transmission of COVID-19** Clinicians (care coordinators and medical staff) to compile risks and discuss with senior team leaders identifying those who may pose a risk including: Risk due to psychiatric characteristics 1. Disorganisation or agitation secondary to mania or psychosis where this involves breach of public health recommendations 2. Risks due to underlying personality characteristics including antisocial traits, including known threats by patient to spread infection to staff or public 3. Cognitive and functional impairment affecting ability to comprehend or abide by public health measures 4. Socio-economic factors effecting transmission 4.1 Unstable accommodation including homelessness, couch surfing 4.2 Living in overcrowded accommodation **Strategies to minimise risk of transmission of COVID-19** 1. Individual risk assessment for each patient based on clinical assessment with above factors 2. Registration and monitoring of risk 2.1 Alert on mental health and general medical patient record (CBIS risk alert) 2.1 Regular review at CMH team meetings **Notification of risks** 1. Establishment of a Mental Health Liaison Officer with network to contact SA Police and Communicable Disease Control Branch (e.g. threats by a patient to harm another with their reported symptoms of COVID-19, or a suspected case reporting they would not adhere to self-isolation)

Overarching this, proactive and responsive leadership was needed to manage usual service demands and COVID-19-specific concerns. This included monitoring and evaluation of incidents via the online Safety Learning System and liaison between service departments and government agencies. Staff health, crucial at a time when staff are anxious about their own health and the health of their families, was supported through practical measures and staff training (Table 5). Responding to staff concerns in a transparent and empathetic fashion was also important to reduce burnout and distress. Further consideration of long-term advocacy and planning for social supports and mental health resources was needed to help meet the anticipated demand in the medium and long term.

**Table 5.** Staff health and training

**Identification of staff at risk (vulnerable staff members)** 1. Advice from the National Cabinet recommends self-isolation for: staff with a compromised immune system or over the age of 70, or over 65 with chronic medical conditions, or over 50 and Aboriginal or Torres Strait Islander people with a chronic medical condition^24^ 2. Staff then to seek advice from their primary health practitioner or specialist regarding their individual level or risk and share this advice confidentially with their manager **Flexible working arrangements** 1. Following sharing medical advice with manager, negotiation of flexible working arrangements including working from home where practicable, use of tele-health, temporary alterations in work duties (such as re-allocation to another team where this may be more possible) 2. Staff living with a vulnerable family member also eligible to explore flexible working arrangements to mitigate this risk 3. If flexible working arrangements are not possible or appropriate for vulnerable staff or staff living with a vulnerable family member, access to COVID-19 special leave with pay, and if this is exhausted, other leave entitlements **Workplace health and safety** 1. Ongoing planning of maintaining social distance in office and vehicles, restricting numbers of staff onsite if required, rotating teams in the office and communication of staff movements 2. MDT and other professional meetings via tele- or videoconference where possible 3. Periodic deep cleans of office and availability of cleaning equipment onsite to maintain cleanliness of environment 4. Availability of PPE 5. Self-assessment and reporting of work health and safety for environments for those working from home **Staff training** 1. Ongoing training and education over videoconference including updates regarding COVID-19 2. SA Health COVID-19 online training for all staff (‘COVID-19 stopping the spread in the workplace’) **Counselling and support for staff during pandemic** 1. Access to Employee Assistance Program (EAP) for counselling 2. Ongoing review of workload management with predicted change in service demands and workforce levels due to sick leave and self-isolation to minimise staff burnout 3. Acknowledgement of high staff anxiety levels in the face of change and uncertainty; compassionate response of leaders and support with work flexibility 3.1 Emphasis on maintaining social support and cohesion within teams, encourage maintaining home routines and exercise, flexibility where possible with duties with consideration to caring responsibilities 3.2 Mindfulness tools emailed daily to all staff to support well-being

## Discussion

Development and implementation of a CMH pandemic response required careful thought and expediency. The rapidly changing situation presented challenges in developing a service plan, necessitating a response providing both structure and adaptability. This needed to be responsive to new concerns, tailored to the local service and individual patients. Risks of exposure and transmission of COVID-19 to vulnerable patients needed to be carefully balanced against risks created by changes in service provision.

As Australia emerges from the pandemic, planning, particularly in regard to potential long-term changes in mental health service delivery, will be critical. Some consequences of the pandemic may be positive. Increased awareness of hygiene could mitigate transmission risks of other infectious diseases, and efficiencies resulting from use of tele-health may improve access for those in remote or inaccessible settings. However, the impact of COVID-19 itself, economic deterioration and consequent effects on lives, livelihoods and families are likely to be significant. Ongoing planning, both in direct mental health service delivery and how best to meet the increasing social need, is crucial.

## Conclusions

The COVID-19 pandemic continues to pose a significant risk to the vulnerable patient population supported by CMH teams in the northern suburbs of South Australia. A public health response document was developed in response to this pandemic. It is hoped this can assist other CMH services in responding to COVID-19, and future pandemics.
